# Pregnancy complicated with Gitelman syndrome: A case report and literature review

**DOI:** 10.1097/MD.0000000000044116

**Published:** 2025-08-29

**Authors:** Liyue Zhang, Xidi Wu, Nan Li, Feifei Huo

**Affiliations:** a Department of Intensive Care Unit, The First Hospital of Jilin University, Changchun, Jilin Province, China.

**Keywords:** Gitelman syndrome, hypokalemia, pregnancy

## Abstract

**Rationale:**

Gitelman syndrome, also known as familial hypokalemia, is primarily characterized by hypokalemic metabolic alkalosis, severe hypomagnesemia, and hypocalciuria.

**Patient concerns:**

A 26-year-old female patient presented with a 4-month history of amenorrhea, intermittent nausea and vomiting for 1.5 months, and worsening symptoms in the past day. She was initially admitted to the reproductive gynecology department with a diagnosis of hyperemesis gravidarum. Due to severe hypokalemia, she was transferred to the ICU.

**Diagnoses:**

Genetic testing revealed 2 heterozygous mutations in the SLC12A3 gene: c.179C>T (p.T60M) and c.1077C>G (p.N359K), confirming the diagnosis of Gitelman syndrome.

**Interventions:**

We gradually decreased the amount of intravenous potassium supplementation, transitioning to oral potassium supplementation. Meanwhile, we administered intermittent magnesium supplementation. This approach maintained the patient’s serum potassium level at approximately 3.0 mmol/L and serum magnesium level at approximately 0.8 mmol/L.

**Outcomes:**

During the hospitalization, a follow-up obstetric ultrasound indicated normal fetal development. The patient was discharged after her condition improved.

**Lessons:**

For pregnant patients with Gitelman syndrome, there is a lack of evidence-based treatment guidelines at present. It is crucial to implement multidisciplinary management and adopt a prudent, individualized approach to enhance the likelihood of achieving favorable maternal and fetal outcomes.

## 1. Introduction

Gitelman syndrome (GS) is an autosomal recessive salt-losing tubular disorder.^[1]^ GS is caused by loss-of-function mutations in the SLC12A3 gene, which encodes the thiazide-sensitive sodium–chloride cotransporter in the renal distal convoluted tubule. These mutations lead to structural and/or functional abnormalities of thiazide-sensitive sodium–chloride cotransporter, impairing sodium and chloride reabsorption in the distal convoluted tubule. This results in a series of pathophysiological and clinical manifestations, including hypovolemia, activation of the renin–angiotensin–aldosterone system, hypokalemia, and metabolic alkalosis.^[2]^

During pregnancy, significant physiological changes occur in women, such as increased blood volume, elevated glomerular filtration rate, and fluctuating hormone levels. These changes may exacerbate electrolyte imbalances in patients with GS and increase the risk of maternal and fetal complications. However, there are few reports in the literature on GS during pregnancy, and no standardized management guidelines are available. The management of pregnant women with GS requires a multidisciplinary approach, involving critical care, obstetrics, endocrinology, nutrition, and other departments. The goal is to maintain electrolyte balance through individualized treatment and to improve maternal and fetal outcomes. Most patients have a good prognosis,^[3–5]^ but some cases of adverse fetal outcomes have been reported.^[6,7]^

This article presents a case of GS in pregnancy, detailing its clinical features, diagnostic process, treatment strategies, and maternal and fetal outcomes. It also discusses the pathophysiological mechanisms, management challenges, and future research directions for GS in pregnancy, with a view to informing clinical practice.

## 2. Case presentation

### 2.1. General information

The patient was a 26-year-old woman, G1P0, admitted to the reproductive gynecology department on August 27, 2024, with “4 months’ amenorrhea, intermittent nausea and vomiting for 1.5 months, worsened in the past day” and diagnosed with “severe morning sickness.” Due to severe hypokalemia, she was transferred to the ICU. She had no significant past medical history. On physical examination, her pulse was 96 beats/min, blood pressure 110/70 mm Hg, and oxygen saturation 100%. No abnormalities were found on cardiopulmonary and abdominal examination, and her limb muscle strength was normal.

### 2.2. Laboratory findings

Serum electrolytes: potassium 1.83 mmol/L (reference range 3.5–5.3 mmol/L), chloride 96.7 mmol/L (reference range 99–110 mmol/L), magnesium 0.26 mmol/L (reference range 0.74–1.02 mmol/L). Arterial blood gas analysis: pH 7.53, PaO_2_ 87 mm Hg, PaCO_2_ 32 mm Hg, bicarbonate 27 mmol/L, base excess 4.0 mmol/L.

### 2.3. Diagnosis and treatment

The patient was clinically diagnosed with early pregnancy, severe morning sickness, electrolyte imbalance (hypokalemia, hypomagnesemia), and metabolic alkalosis. A central venous catheter was placed, and continuous potassium chloride infusion was initiated. She also received intravenous nutrition, antiemetic therapy, and magnesium supplementation. Initially, she required approximately 24 g of potassium daily, and it took a week to stabilize her serum potassium around 3.5 mmol/L.

**Figure 1.**
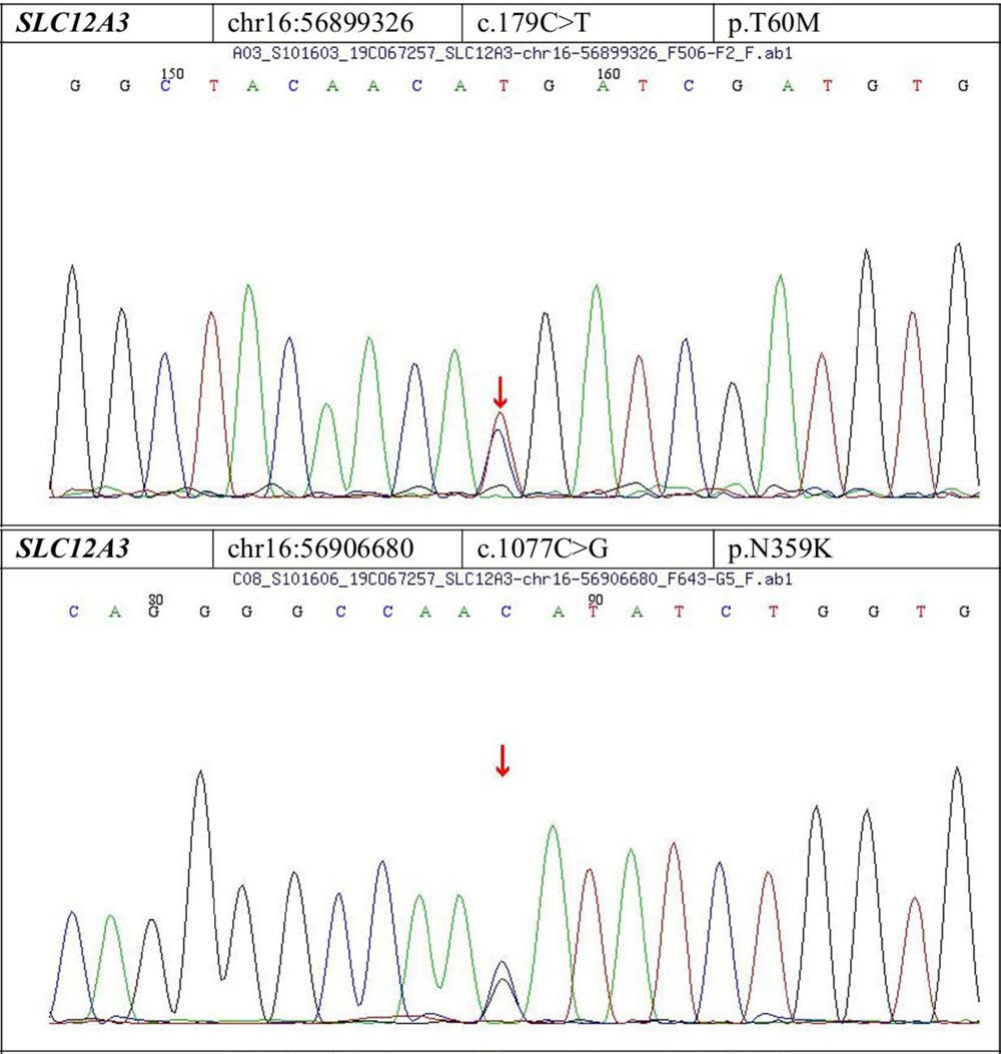
Two heterozygous mutations in the SLC12A3 gene.

The unusually high potassium requirement prompted further evaluation. Considering her hypomagnesemia and metabolic alkalosis, GS was suspected. Additional tests revealed: 24-hour urine potassium 312.6 mmol/24 h (reference 51–102), sodium 263.8 mmol/24 h (reference 130–260), calcium 0.94 mmol/24 h (reference 2.5–7.5), chloride 539 mmol/24 h (reference 100–250), and phosphorus 9.6 mmol/24 h (reference 22–48). Genetic testing identified 2 heterozygous mutations in the SLC12A3 gene: c.179C>T (p.T60M) and c.1077C>G (p.N359K) (Fig. 1). Family analysis showed the mother had the c.179C>T mutation, and the father had the c.1077C>G mutation. This confirmed the GS diagnosis.

Treatment was adjusted to reduce intravenous potassium, transitioning to oral supplementation, while continuing intermittent magnesium. Serum potassium was maintained around 3.0 mmol/L and magnesium around 0.8 mmol/L. During hospitalization, obstetric ultrasound confirmed normal fetal development. The patient improved and was discharged on September 20.

## 3. Discussion

GS, a benign tubular disorder with a prevalence of 1/40,000 to 10/40,000 (possibly higher in Asia), typically manifests in adolescence or adulthood but can also occur in children.^[8]^ Clinical features include muscle weakness, fatigue, exercise intolerance, syncope, tetany, paresthesia, growth delay, delayed puberty, and short stature. Typical biochemical findings are metabolic alkalosis and the “five lows and one high”: hypokalemia, hypomagnesemia, hypochloremia, hypocalciuria, hypotension, and heightened renin–angiotensin–aldosterone system activity.

In this case, the patient’s severe morning sickness exacerbated hypokalemia. Pregnancy increases potassium requirements and glomerular filtration rate,^[9,10]^ leading to greater sodium and electrolyte loss. Elevated aldosterone levels during pregnancy further enhance potassium and magnesium excretion. These factors can easily mask GS, necessitating suspicion of GS in refractory hypokalemia cases. Further evaluation with serum magnesium and 24-hour urine electrolytes is recommended, along with genetic testing where available.

Treatment involves individualized, lifelong oral potassium and/or magnesium supplementation. Hypomagnesemia should be addressed first, as magnesium supplementation aids potassium repletion and reduces complications like tetany.^[11,12]^ Target serum levels are 3.0 mmol/L for potassium and 0.6 mmol/L for magnesium. However, achieving these targets can be challenging and may cause side effects like vomiting, diarrhea, or gastric ulcers. A balance between target levels and drug tolerability is essential. Potassium supplements should be chloride-based (KCl) to address chloride loss. Initial oral dose is 40 mmol KCl (1–2 mmol/kg in children). Intravenous KCl is reserved for severe cases with complications like arrhythmias or paralysis.^[13]^ KCl should be diluted in saline, with concentrations up to 40 mmol/L peripherally and up to 80 mmol/L centrally, at a maximum rate of 20 mmol/h.

Magnesium deficiency exacerbates hypokalemia.^[14]^ Oral magnesium supplementation is preferred for hypomagnesemia. Elemental magnesium starting at 300 mg/d (5 mg/kg in children), divided into 2 to 4 doses with meals, is recommended. Doses should be adjusted based on serum magnesium and gastrointestinal tolerance. Intravenous magnesium is used for acute severe hypomagnesemia or intolerance to oral supplements.

In pregnancy, early treatment planning for hypokalemia and hypomagnesemia is crucial, with adjustments as needed. If potassium supplementation is ineffective or poorly tolerated, potassium-sparing diuretics like spironolactone (starting at 100 mg/d, up to 300 mg/d) can be considered. Spironolactone is a Category C drug in pregnancy, but some reports show no significant complications in GS patients.^[15,16]^ Alternatives include eplerenone and amiloride.^[17,18]^ Eplerenone, with fewer endocrine side effects than spironolactone, starts at 50 mg/d (up to 150 mg/d), while amiloride starts at 10 mg/d (up to 20–30 mg/d). Both are category B drugs. A randomized crossover trial in 30 GS patients showed that adding eplerenone (150 mg/d) or amiloride (20 mg/d) to electrolyte supplementation increased serum potassium by 0.15 and 0.19 mEq/L, respectively, with 10% achieving normal potassium levels.^[19]^

Normalization of electrolytes is often unattainable in GS patients, especially during pregnancy. Basu et al suggest that in pregnant GS patients, the goal is to prevent adverse delivery outcomes like seizures or spasms rather than normalizing potassium and magnesium levels.^[20]^ In this case, oral potassium supplementation maintained serum potassium around 3.0 mmol/L without clinical hypokalemia, achieving treatment goals.

## 4. Conclusion

In summary, there is a lack of evidence-based guidelines for managing GS in pregnancy. Given the nonspecific clinical presentation of GS, multidisciplinary management is essential. Individualized treatment and close electrolyte monitoring are crucial to preventing complications and achieving favorable maternal and fetal outcomes.

## Author contributions

**Investigation:** Xidi Wu, Nan Li.

**Writing – original draft:** Liyue Zhang.

**Writing – review & editing:** Feifei Huo.
